# Comparison of Lifestyles of Women With Gestational Diabetes and Healthy Pregnant Women

**DOI:** 10.5539/gjhs.v7n2p162

**Published:** 2014-10-09

**Authors:** Fatereh Momeni Javid, Masoumeh Simbar, Mahrokh Dolatian, Hamid Alavi Majd

**Affiliations:** 1 Department of Reproductive Health and Midwifery, Shahid Beheshti University of Medical Sciences, Tehran, Iran; 2 Research Center for Safe Motherhood (RCSM), Department of Reproductive Health and Midwifery, Faculty of Nursing and Midwifery, Shahid Beheshti University of Medical Sciences, Tehran, Iran; 3 Department of Reproductive Health and Midwifery, Faculty of Nursing and Midwifery, Shahid Beheshti University of Medical Sciences, Tehran, Iran; 4 Department of Biostatics, School of Paramedical Sciences, Shahid Beheshti University of Medical Sciences, Tehran, Iran

**Keywords:** lifestyle, gestational diabetes, diet during pregnancy, self-care, perceived stress

## Abstract

**Background:**

Gestational diabetes is the most common medical condition in pregnancy and can be a predisposing factor in incidence of type II diabetes in future. Incorrect lifestyles can predispose people to various diseases, including diabetes, which is a serious health risk. Therefore, this study was conducted to compare lifestyles of women with gestational diabetes and healthy pregnant women attending the health centers affiliated to Shahid Beheshti University of Medical Sciences (SBMU) in 2013.

**Methods:**

A comparative-descriptive study was conducted on 100 pregnant women with definite diagnosis of gestational diabetes and 100 healthy pregnant women attending the health centers affiliated to SBMU. Data were collected by a researcher-made questionnaire about lifestyle during pregnancy. Informed consents were obtained, and sampling was performed using convenient multi-stage random sampling method. Data were analyzed using SPSS-18 software.

**Results:**

Demographic and obstetrics variables were not significantly different in the two groups. However, two groups were significantly different regarding their lifestyles as the mean scores of pregnancy diet were 65.05 in the diabetic group and 74.12 in the healthy group (P<0.001), and the mean moderate physical activity scores in the two groups were 4.62 and 5.69, respectively (P=0.042), the mean pregnancy self-care scores were 71.9 and 81.87, respectively (P<0.001), the mean perceived social support in the two groups were 65.75 and 73.88, respectively (P<0.001), and the mean scores of perceived stress were 51.57 and 60.27, respectively (P<0.001).

**Conclusion:**

Women’s lifestyles were different in some areas. This study further reveals the importance of women’s diet, self-care, moderate physical activity, and perceived social support during pregnancy. Incidence of gestational diabetes can be prevented through increased awareness and education of pregnant women about having appropriate lifestyles during pregnancy and any intervention that would lead to improved lifestyle.

## 1. Introduction

Gestational diabetes is defined as varying degrees of carbohydrate intolerance, first begun or diagnosed during pregnancy (Cunningham et al., 2010). Gestational diabetes is increasing globally (Bener, Saleh, & Al-Hamaq, 2011). Nearly 1% to 14% of pregnancies are complicated by diabetes, of which, 3% to 5% are gestational diabetes (Ferrara, 2007; Nilsson, 2013; Ricci, 2009). In a meta-analytical study, the prevalence of gestational diabetes in Iran was estimated at 4.9%. The prevalence of gestational diabetes in Tehran is 6.9%, and varies in other parts of the country (Sayehmiri et al., 2013). Generally, the prevalence of gestational diabetes depends on the study population and use of different diagnostic criteria (Halperin & Feig, 2014).

More than 50% of women with gestational diabetes will have type II diabetes in the next 20 years (Cunningham et al., 2010; Ferrara, 2007). Since pregnancies complicated by diabetes require increased health care resources to control sugar levels during pregnancy and reduce severe perinatal consequences, increasing prevalence of gestational diabetes has caused serious concerns for health systems worldwide (Ferrara, 2007).

Pregnancy has been described as a critical period. With the beginning of pregnancy, mother’s body go through many changes that turn her into a new person with new physical and mental characteristics, which lead to changes in health behavior and lifestyle (Moshki, Bahri, & Sadegh Moghadam, 2012; Zolfaghari, 2007). Therefore, it seems choice of lifestyle during pregnancy can have lasting and long-term effects on health of the mother and infant (Fraser & Cooper, 2003).

In the 21^st^ century, we are witnessing industrialization, globalization, increased life expectancy, and changes in lifestyles of people around the world. One of the consequences of these changes is changes in disease pattern and the prevalence of chronic diseases, like diabetes (Shojaeezadeh, Estebsari, Azam, & Batebi, 2008). Inappropriate lifestyle can lead to the incidence of various diseases, including diabetes, which is a serious health risk (Mansoorian et al., 2012). Proper lifestyle is so important that a new branch has been created in medical sciences called “lifestyle medicine”, with applications in control and prevention of diseases. Lifestyle is associated with the person’s daily life patterns such as diet and food habits, leisure time, smoking habit, physical activity, stress, and normal use of health care services (Shojaeezadeh et al., 2008).

Most studies have been conducted on diabetes type II, and few factors have been investigated in this regard. We did not find any studies on gestational diabetes with the approach to factors. Gestational diabetes is one of the most prevalent medical complications in social determinants of health during pregnancy and can be a predisposing factor for diabetes type II in future. Given all these, methods can be devised to improve lifestyle during pregnancy and prevent gestational diabetes from social determinants of health perspective by recognizing the differences in lifestyles of Iranian pregnant women with and without diabetes. This study, therefore, was conducted to determine and compare the lifestyles of women with gestational diabetes and healthy pregnant women attending the health centers affiliated to Shahid Beheshti University of Medical Sciences (SBMU) in 2013.

## 2. Methods

This comparative descriptive study was conducted on 200 pregnant women, comprising 100 women with definite diagnosis of gestational diabetes (diseased group), and 100 healthy pregnant women (healthy group) that attended obstetrics clinics in hospitals affiliated to Shahid Beheshti University of Medical Sciences in 2013. In this study, sampling was performed using multi-stage random sampling method. In the first stage, a number of centers were randomly selected, and each was considered as a cluster. In the second stage, sampling from each selected center continued until target sample size was reached.

Study inclusion criteria were Iranian nationality, positive glucose challenge test (GCT) (for the diabetic group), aged between 18 and 35 years, gestational age between 24 and 28 weeks based on accurate and reliable first day of last menstruation, or the first trimester ultrasound, singleton pregnancy, less than 4 pregnancies, no history of macrosomia (birth weight more than 4000 grams) in previous pregnancies, no history of neonatal mortality in previous pregnancies, no history of infant or embryo malformation in previous pregnancies, no previous gestational diabetes, no family history of type II diabetes, no history of frequent miscarriages more than twice, no history of infertility or known polycystic ovary syndrome, no obesity (BMI>30), no known chronic diseases (hypertension, cardiovascular diseases, kidney diseases, anemia, thyroid, and autoimmune diseases).

In this study, data were collected using form 1 for entry criteria and blood sugar check-list, and form 2 containing demographic and obstetrics details, and a researcher-made lifestyle questionnaire including: diet, physical activity, self-care in pregnancy, perceived stress, and perceived social support.

Diet questionnaire contained 20 items with 3-point Likert scale with maximum score of 60 and minimum of zero. Validity and reliability of the questionnaires were assessed in the present study.

Physical activity was assessed using Pregnancy Physical Activity Questionnaire, in which type of activity is divided into three groups of exercise, activity at home, and in leisure time, in the number of hours per day and per week. Intensity of physical activity was measured in Metabolic Equivalent Test (MET). To calculate the intensity of activity, value of MET is multiplied by duration of activity per day per week. Activity rate, based on type of activity, is the sum of intensity per day or per week. Activity with MET value less than 1.5 is considered sedentary, between 1.5 and less than 3 is considered light, between 3 and 6 moderate and more than 6 intense. Validity of this questionnaire was confirmed by Chasan-Taber et al. (2004), and its reliability was confirmed in a study by Kazemi and Ahmadi (2007) with Cronbach’s alpha of 0.85.

The researcher-made prenatal self-care questionnaire contained 13 items, and scored according to 4-point Likert scale, from “never” (zero points) to “always” (3 points). Thus, minimum score was zero and maximum 39. Its validity and reliability was assessed in the present study.

Cohen’s perceived stress scale was used to assess general perceived stress in the past month, including thoughts and feelings about stressful events, control, overcoming, and coping with stress. In this study, the 14 item version of the questionnaire was used. The 7 negative items indicated inability to cope with stress, and 7 positive items showed a well-adjusted person to stressful factors. Scoring was based on the 5-point Likert scales, from “never” (zero points) to “many occasions” (4 points) (Leung, Lam, & Chan, 2010). Therefore, the lowest score is zero and the highest 56, and higher scores indicate more perceived stress. Reliability of the Persian version was assessed by Bastani et al. (2008) using internal consistency method, and Cronbach’s alpha was found 0.74 (Bastani et al., 2008). Other studies have confirmed the reliability of the questionnaire with Cronbach’s alpha of 0.81 using internal consistency (Mazlom, Vaghei, Modarres, Kashani Lotfabadi, & Shad, 2012).

Perceived social support scale was designed by Saracen et al. in 1983, and contains 12 items in Likert scale, from “totally disagree” (zero points) to “totally agree” (6 points). To make the above variables comparable, scores were converted into 0 to 100 scale. Accordingly, 0-33 was considered unfavorable, 34-66 moderate and 67-100 favorable. The internal consistency was calculated at Cronbach’s alpha of 0.97, Naseh et al. (2012) converted the questionnaire in to Persian, and its reliability was found at Cronbach’s alpha of 0.95 (Naseh et al., 2012).

Considering that these questionnaires were used by pregnant women, validity and reliability of each was evaluated again in the present study. To assess validity, two quantitative content validity methods were assessed, including Content Validity Ratio (CVR) and Content Validity Index (CVI). In the present study, CVR was found 0.92 for dietary style, 0.85 for physical activity, 0.83for prenatal self-care, 0.86 for perceived social support, and 0.95 for perceived stress. Also, CVI was found 0.92 for dietary style, 0.88 for physical activity, 0.91 for prenatal self-care, 0.99 for perceived social support, and 0.9 for perceived stress. In this study, CVR and CVI for each lifestyle domain and every single item was found to be at an acceptable level, and no question had to be eliminated.

Reliability of the questionnaires was measured using internal consistency and test-retest methods.

A) Internal consistency method: In a pilot study, the questionnaires were completed by 30 pregnant women and data obtained were analyzed. Cronbach’s alpha coefficient for the whole lifestyle questionnaire was found 0.85.
B) Test-retest method: 15 pregnant women were selected for the internal consistency method, completed the questionnaire again after 12 days. Spearman correlation coefficient was calculated between scores of two completion procedures. Spearman correlation coefficient was r=0.91 for the whole lifestyle questionnaire (0.85 for dietary style, 0.99 for physical activity, 0.92 for self-care, 0.97 for perceived social support, and 0.95 for perceived stress).

After obtaining written consent, sampling was carried out in health centers. Eligible women that met study inclusion criteria according to their blood sugar level entered the study. Women were divided into two groups of gestational diabetic group and healthy group according to their blood sugar level and diagnosis of gestational diabetes in weeks 24-28. Study objectives and methods were explained to study subjects, and data forms including demographic and obstetrics details and also lifestyle questionnaire were completed using interview technique, and women were studied in terms of the above variables from the beginning of pregnancy to the 28^th^ week (prior to diagnosis of gestational diabetes).

Data were analyzed using SPSS-18 software. Descriptive statistics were used to present frequency, percentage, mean, and standard deviation in tables, and inferential statistics were used for analysis of data. To find relationships, statistical tests including independent t-test, Chi-square test, odds ratio, and Mann-Whitney test were used, as well as multivariate logistic regression to compare independent variables in the two groups.

## 3. Results

Comparison showed no significant differences between the two groups in terms of potentially confounding variables in incidence of gestational diabetes including age, number of pregnancies, parity, gestational age, abortions, and socioeconomic factors including education level, education level of spouse, occupation, spouse’s occupation, income, and home-ownership. The results showed no significant differences between the two groups in terms of obstetrics variables such as age at first pregnancy, preconception care, and gestational age at first care. However, there was a significant difference between the two groups in terms of participation in delivery preparation classes and water exercises (Table 1).

**Table 1:** Demographic and reproductive history of women with (Gestational Diabetes Mellitus) GDM and Non GDM

Variable		GDM (n=100)		Non GDM (n==100)		P	Test
		**Mean**	**SD**	**Mean**	**SD**		
**Age (year)**		28.68	3.835	27.8	4.569	0.181	
**Gestational Age in first care(week)**		5.88	2.679	5.31	2.953	0.154	
**BMI pre pregnancy *(kgm^2^)***		23.83	2.940	23.90	3.350	0.866	
		**N**	**%**	**N**	**%**	**P**	
**Educational**	Illiterate	1	1	1	1	0.773	
	Primary school	3	3	3	3		
	Middle school	25	25	14	14		0.773	
	High school	40	40	58	58		0.773	
	University	31	31	24	24		0.773	
**Occupational**	Householder	86	86	92	92	0.175	
	Employed	14	14	8	8		
**Accommodation ownership**	Personal	38	38	39	39		
	Rental	62	62	61	61	0.884	
**Income**	<500000	18	18	9	9	0.184	
	500000-1000000	59	59	65	65		
	>1000000	23	23	26	26		
**Gravida**	1	43	43	50	50	0.411	
	2	38	38	32	32		
	3	16	16	15	15		
	4	3	3	3	3		
**Gestational Age(week)**	24-27	12	12	9	9	0.584	
	28-31	25	25	24	24		
	32-35	34	34	37	37		
	36-40	29	29	30	30		
**Miscarriage History**	Yes	26	26	23	23	0.622	
	No	74	74	77	77		
**Para**	0	57	57	62	62	0.563	
	1	34	34	28	28		
	2	9	9	10	10		
**Pre conception**	Yes	27	27	30	30	0.638	
	No	73	73	70	70		
**Delivery preparation classes**	Yes	13	13	26	26	0.020	
	No	87	87	74	74		
**Aqua gymnastic**	Yes	3	3	11	11	0.027	
	No	97	97	89	89		

SD= Standard Deviation.

**Table 2:** Frequency and comparison of lifestyles’ mean-scores of women with GDM and Non GDM

Lifestyles Item		Non GDM (n=100) M(SD)	GDM (n=100) M(SD)	P-value
Nutrition Style		65.05(9.68)	74.12 (9/90)	<0.001
Physical Activity(MET)	Sedentary=leisure activity(<1.5)	5.64(2.712)	4.70(3.747)	0.223
	Light activity=home activity(1.5-<3)	9.07(5.524)	10.75(6.886)	0.059
	Moderate activity=exercise(3-6)	4.62(7.520)	5.69(4.469)	0.042
Self-Care in pregnancy		71.90(11.484)	81.87(7.542)	<0.001
Perceived Social Support		65.75(14.626)	73.88(15.228)	<0.001
Perceived Stress		51.57(11.464)	60.27(13.126)	<0.001

**Table 3:** Logistic regression model of effective factors on gestational diabetes

Group Factor	P	OR	CI (95%)
Age	0.098	1.08	0.986-1.187
Gravid	0.737	0.92	0.569-1.491
delivery preparation classes	0.548	0.73	0.268-2.014
Aqua gymnastic	0.721	0.71	0.111-4.580
BMI pre pregnancy	0.908	1.00	0.898-1.129
Nutrition style	0.008	0.94	0.913-0.986
Physical activity	0.285	0.98	0.956-1.013
Self-Care in pregnancy	0.001	0.93	0.893-0.970
Perceived Social Support	0.770	0.99	0.972-1.021
Perceived Stress	0.040	0.96	0.941-0.999

OR= Odds Ratio; CI=confidence.

According to logistic regression model, dietary style, prenatal self-care and perceived stress significantly affected the incidence of gestational diabetes. This means that for every unit increase in score of dietary style, the odds of gestational diabetes reduced by 6%, and since dietary style score ranged 0-100, for every 10 units increase in score of dietary style, odds of gestational diabetes reduced by 60%, which shows more attention should be paid to dietary style of pregnant women to prevent gestational diabetes. It was also found that for every unit increase in self-care, odds of gestational diabetes reduced by 7%. Given the self-care score range of 0-100, for every 10 units of increase in self-care score, odds of gestational diabetes reduced by 70%. It was shown in this model that for every unit increase in perceived stress, odds of gestational diabetes reduced by 4%.

## 4. Discussion

Investigating lifestyle by considering social determinants of health in incidence of gestational diabetes distinguishes the present study from others. The results showed a significant difference between the two groups in dietary style during pregnancy. In the present study, most women in the diabetic group (55%) were moderate quality in terms of dietary style. A study showed that use of western style nutritional pattern was associated with increased risk of gestational diabetes (Akhondan, Mirmiran, Rashidkhani, & Asghari, 2011). Another study showed that constant use of high fiber fruit and vegetable nutritional regimen before pregnancy significantly reduces risk of gestational diabetes, so that increased absorption of 10 grams of fiber per day is associated with 26% reduction in risk of gestational diabetes. Conversely, intake of high glycemic index nutrition is positively associated with risk of gestational diabetes (Zhang, Liu, Solomon, & Hu, 2006). The results of other studies are in line with those of the present study, which show necessity of attention to dietary style of pregnant women, thus, educational and counseling interventions in this area are recommended.

Mean duration of <1.5 MET activity and low intensity activity were not different between the two groups. However, mean duration of moderate intensity activity was significantly higher in the healthy group than in the diabetic group. Casual moderate and intense physical activity according to MET was associated with 27% reduction in risk of increased blood sugar level in pregnancy. Beneficial biological effects of physical activity on blood sugar metabolism and sensitivity to insulin in non-pregnant women have clearly been demonstrated. Participation in physical activities reduces blood sugar concentration, increases sensitivity to insulin, improves cardiovascular system, and reduces body fat. There is evidence that indicates these beneficial effects on sensitivity to insulin and beta cells happen during pregnancy, too (Deierlein, Siega-Riz, & Evenson, 2012).

Physical activity is also associated with modification of lipid density, insulin sensitivity of cell, reduced lipid mass, reduced cytokines before inflammation, and C-reactive protein in peripheral blood circulation, which can correct impaired endothelial function (Sorensen et al., 2003). In a meta-analysis, it was found that consistent physical activity before pregnancy or in early pregnancy can potentially prevent progress of gestational diabetes (Tobias, Zhang, van Dam, Bowers, & Hu, 2011).

Moreover, a clinical trial conducted as an exercise program for obese and overweight pregnant women exposed to risk of gestational diabetes in the second and the third trimesters showed that exercise intervention during this period of pregnancy had no effect on fasting blood sugar level, sensitivity to insulin, or birth weight, which was probably due to improper performance of physical exercises during the study (Oostdam, van Poppel, Eekhoff, Wouters, & van Mechelen, 2009). It appears that mothers’ concerns about exercise during pregnancy are associated with their lack of knowledge about permitted exercises and how they should be performed. Since incorrect information affects their behaviors, it can lead to sedentary lifestyle during pregnancy (Noohi, Nazemzadeh, & Nakhei, 2010). Considering that most pregnant women believe that they are not allowed to do physical exercises during pregnancy, or they lack information about how much and how they should perform these activities, the present study results in this area emphasize the necessity for more attention of healthcare personnel, especially midwives, regarding consultation before pregnancy and prenatal care to provide clients with sufficient and correct information about conditions of performing physical activities during pregnancy.

The present study results indicate that level of prenatal self-care was different in the two groups. In a study on people with diabetes, it was found that people with better self-care had lower fasting blood sugar. Accordingly, appropriate self-care behaviors in patients with diabetes leads to better control of their blood sugar level, and also patients with better self-care had lower Body Mass Index (Jafarian, Zabihi, Babaieasl, Eshkevari, & Bijani, 2010). In the present study, two groups were significantly different regarding their participation in the delivery preparation classes and timely pregnancy care as the items of self- care. Review of various studies showed that participation in delivery preparation classes and receiving group training led to mothers’ using each other’s experiences, which in addition to reducing their anxiety, it can also play a significant role in reducing diseases, reducing complications, and improving their health through increased knowledge and skills in this period (Toughyani, Ramezani, Izadi, & Motie, 2008). In pregnancy classes, neuro-muscular, moderate physical, and relaxation exercises are also performed in addition to educational issues, which can reduce pregnancy complaints and increase daily activities of pregnant women (Mehdizadeh, Roosta, Kamali, & Khoshgoo, 2003).

In the present study, the mean score of perceived social support in healthy pregnant women was significantly higher than that in the group with gestational diabetes. A study showed that perceived social support from the family is significantly associated with control of type II diabetes (Carcone, Ellis, Weisz, & Naar-King, 2011). People that enjoy social support have better quality of life (Chan, Hon, Chien, & Lopez, 2004). In a study, lack of social support was introduced as an important risk factor to mother’s health during pregnancy, which leaves damaging effects on pregnancy outcomes (Elsenbruch et al., 2007).

Study results showed that mean score of perceived stress in the group with gestational diabetes was lower than that in in the healthy pregnant women group. Although psychosocial stress positively affects risk of type II diabetes, there are few studies in pregnancy period. In a prospective cohort study with the aim to investigate the relationship between perceived stress during pregnancy and incidence of gestational diabetes on 1114 Spanish pregnant women, it was found that psychosocial stress in study population had no significant relationship with incidence of gestational diabetes. Also, after adjustments for confounding factors (age and BMI) in a group of women with severe pregnancy stress compared to women with low pregnancy stress, no increase was observed in risk of gestational diabetes (Silveira, 2013).

## 5. Conclusion

Finally, study results showed that lifestyle of women with gestational diabetes was different from that of healthy pregnant women, so that dietary style, physical activity, prenatal self-care, perceived social support, and perceived stress were more favorable in healthy pregnant women, which is indicative of the need for attention to these issues during pregnancy. Incidence of gestational diabetes can be prevented through increased awareness and education of pregnant women about having appropriate lifestyles during pregnancy and any intervention that would lead to improved lifestyle.
